# Modeling and Simulation of Procoagulant Circulating Tumor Cells in Flow

**DOI:** 10.3389/fonc.2012.00108

**Published:** 2012-09-14

**Authors:** Angela M. Lee, Garth W. Tormoen, Eva Kanso, Owen J. T. McCarty, Paul K. Newton

**Affiliations:** ^1^Department of Aerospace and Mechanical Engineering, Viterbi School of Engineering, University of Southern CaliforniaLos Angeles, CA, USA; ^2^Department of Biomedical Engineering, School of Medicine, Oregon Health and Science UniversityPortland, OR, USA; ^3^Department of Cell and Developmental Biology, School of Medicine, Oregon Health and Science UniversityPortland, OR, USA

**Keywords:** procoagulant circulating tumor cells, circulating tumor cells, chemical gradient tracking, tissue factor and coagulation, prothrombin and thrombin fields, circulating tumor cell induced hypercoagulation

## Abstract

We describe a mathematical/computational model for thrombin concentration gradients generated by procoagulant circulating tumor cells (CTCs) in flow. We examine how CTCs enhance blood coagulation as they diffuse tissue factor-dependent coagulation enzymes in a flow environment with vessel walls. Concentration fields of various enzymes, such as prothrombin and thrombin, diffuse, to, and from CTCs, respectively, as they propagate through the bloodstream. The diffusion-dependent generation of these enzymes sets up complex time-dependent concentration fields. The CTCs are modeled as diffusing point particles in an incompressible fluid, and we exploit exact analytical solutions based on three-dimensional Green’s functions for unbounded domains with one wall for high resolution numerical simulations. Time-dependent gradient trackers are used to highlight that concentration fields build-up (i) near boundaries (vessel walls), (ii) in regions surrounding the diffusing particles, and (iii) in complex time-dependent regions of the flow where fields associated with different particles overlap. Two flow conditions are modeled: no flow, and unidirectional constant flow. Our results indicate that the CTC-generated thrombin diffuses to and persists at the blood vessel wall, and that the spatial distribution of CTCs in flow determines local thrombin concentration. The magnitude of the diffusion gradient and local thrombin concentration is dependent upon bulk solution concentrations of coagulation factors within normal reported concentration ranges. Therefore, our model highlights the potential to determine patient-specific risks for CTC-induced hypercoagulability as a function of CTC number and individual patient concentration of coagulation factors.

## Introduction

Metastatic cancer accounts for the majority of deaths caused by cancer. Metastasis is believed to result from tumor cells from a primary site, migrating toward and intravasating into a blood vessel, navigating the blood circulation to arrive at a distant site whereby it arrests from the blood flow, extravasates, and establishes a metastatic tumor site. The process of metastasis thereby exposes a tumor cell to a variety of new environments, and poses significant physical challenges the tumor cell must overcome if it is to successfully metastasize.

The interactions between circulating tumor cells (CTCs) and blood coagulation proteins have not been fully characterized. Activation of the blood’s coagulation system has been associated with cancer, particularly metastatic cancer, for centuries (Gay and Felding-Habermann, 2011). The exact mechanism(s) underlying the activation of blood coagulation in cancer remain ill-defined (Khorana et al., 2007; Gay and Felding-Habermann, 2011). Tumor cell expression of tissue factor (TF) has been associated with advancing stages of cancer progression, and has been shown to correlate with metastatic potential *in vivo* (Mueller et al., 1992; Mueller and Ruf, 1998; Amirkhosravi et al., 2002). TF is a transmembrane glycoprotein that is normally expressed by cells outside of the blood vasculature. The exposure of blood to TF, as occurs in the event of a blood vessel injury, is a physiological initiator of coagulation (Gomez and McVey, 2006; Okorie et al., 2008). TF serves as the cell membrane receptor for and enzyme cofactor of coagulation factor VIIa (FVIIa). In complex, TF-FVIIa activate the extrinsic pathway of coagulation leading to the formation of thrombin which can then convert fibrinogen to fibrin in order to form a plug that stops bleeding at the injury site in order to maintain blood flow and volume.

In the context of a metastasizing tumor cell, a TF-expressing CTC may expose blood within an uninjured blood vessel to TF (Versteeg et al., 2004; Khorana et al., 2007; Berny-Lang et al., 2011; Otero et al., 2011; Tormoen et al., 2011). Levels of intravascular TF correlate with cancer progression and to some extent with the formation of pathological clots or thrombi in the veins of patients with cancer. Thrombosis, the formation of pathological thrombi, accounts for the second leading cause of death for patients with cancer and constitutes a significant source of morbidity in these patients (Versteeg et al., 2004). Anticoagulant measures taken after a thrombotic event are effective at reducing the formation of subsequent thrombi, but no current laboratory assay is capable of predicting which patients are at risk to develop thrombosis. The incidence of thrombosis is known to correlate with cancer type and tissue of origin, suggesting that the cancerous cells themselves have a role in the formation of pathological thrombi (Blom et al., 2005). *In vitro*, cancer cells are capable of independently initiating coagulation and clotting blood plasma. Similarly, functionally blocking TF on cancer cells prevents the cell’s ability to clot blood plasma. Therefore, mounting evidence suggests that cancer cell expressed TF is a likely culprit for the initiation of blood coagulation associated with cancer (Berny-Lang et al., 2011; Marchetti et al., 2011, 2012; Saito et al., 2011; Yates et al., 2011; Welsh et al., 2012).

The ability of TF to activate blood coagulation is dependent upon the presence of phospholipids, suggesting that only cell surface-expressed TF or cell membrane-derived TF bearing microvesicles are capable of activating coagulation (Nemerson, 1968). This also indicates that the activation of coagulation by TF is essentially a surface phenomenon, requiring coagulation factors to transport from the blood to the surface-expressed TF in order to participate in coagulation. *In vitro*, the trafficking of soluble coagulation factors from bulk to a TF-expressing phospholipid surface is rate-limiting with respect to enzyme activation (Gemmell et al., 1988; McGee et al., 1992; Hall et al., 1998). Further, TF activity is augmented in the presence of blood flow where convective transport supplants diffusive transport as the dominant mode of transport for coagulation factors to TF. Taken together, the ability for a TF-expressing CTC to activate blood coagulation is likely dependent upon its spatio-temporal relationship with the blood. *In vitro*, the coagulation kinetics for cancer cells in suspension is dependent upon the number of cells added to plasma (Berny-Lang et al., 2011; Tormoen et al., 2011; Yates et al., 2011; Welsh et al., 2012). Further work has suggested that TF-expressing cells in suspension show synergistic effects on their ability to initiate and propagate coagulation, with the time to initiate coagulation enzymes, and the rate at which these enzymes are generated correlating with the average separation distance between cells rather than the overall cell count (Tormoen et al., 2011). The effects of spatial separation on coagulation kinetics are consistent in assays that utilize closed systems under well-mixed conditions as well as open systems under laminar flow. However, a CTC would experience different flow regimes if it were circulating on the arterial side versus the venous side or if it were free-flowing or adherent to the cell wall, and the effects that these different conditions have on coagulation kinetics have not been established.

In this manuscript, we model the concentration of thrombin generated by dispersed CTCs under constant laminar flow. Our model is based upon exact solutions used in the atmospheric dispersion community (Stockie, 2011) whereby a source of pollution near the ground (i.e., a smokestack) emits a pollutant which enters the atmosphere and is dispersed and diffused downstream as a “Gaussian plume” or a “Gaussian puff” (Stockie, 2011). We adapt and use the solutions, which are based on a Green’s function formulation for the concentration field equations (Kevorkian, 1989), to model the dispersing and diffusing thrombin concentration field entering the blood. Since the concentration field equations are linear, we can superpose as many fields from each of the CTCs as needed. We assume that the transport of coagulation factors is diffusion-limited as viscous forces dominate inertial forces of the cells. Our results, obtained in a simple geometry with a single (vessel) wall, suggest that thrombin generated by a CTC collects at the blood vessel wall and correlates with the number and spatial distribution of CTCs in the blood, supporting a role for the CTC count in predicting risk for developing thrombosis. Modeling and simulation of coagulation processes has been performed (Fogelson and Tania, 2005; Fogelson, 2007; Bodnar and Sequeira, 2008; Chatterjee et al., 2010; Gregg, 2010; Leiderman and Fogelson, 2011), but to our knowledge, our work is the first to model the physiologically relevant scenario of a TF-expressing cell entering into and circulating within the bloodstream and simulate its effects on coagulation processes.

## Materials and Methods

### Fluid dynamics and concentration field equations

The computational model is based on the partial differential equations for the diffusing concentration field, coupled with the equations for incompressible fluid flow (Pedley, 1980; Kevorkian, 1989):

(1) $$\frac{\partial {\vec{c}}^{\left(i\right)}}{\partial t}+\vec{u}\cdot \nabla {\vec{c}}^{\left(i\right)}=\alpha_{i}\Delta {\vec{c}}^{\left(i\right)}+\delta \left(\vec{x}-{\vec{x}}_{i}\right)$$

(2) $${\dot{\vec{x}}}_{i}=\vec{u}$$

(3) $$\nabla \cdot \vec{u}=0$$

(4) $$\rho \left(\frac{\partial \vec{u}}{\partial t}+\vec{u}\cdot \nabla \vec{u}\right)=-\nabla p+v\Delta \vec{u}$$

(5) $$\vec{u}\left(\vec{x},0\right)=\vec{f}\left(\vec{x}\right)$$

Here, the concentration field associated with the *i*th species is denoted ${\vec{c}}^{\left(i\right)}\left(x,y,z;\;t\right),$ with diffusion coefficient α*_i_*. The fluid (blood) velocity field is denoted by $\vec{u}\left(\vec{x},\;t\right),$ blood pressure denoted *p*, density ρ, and each of the “*i*” CTCs (*i* = 1, …, *n*) are located at ${\vec{x}}_{i}\left(t\right),$ their time derivatives are denoted by the “dot” superscript. δ in Eq. 1 is the Dirac-delta function which is zero everywhere but where the argument is zero, which in this case are the locations of each of the CTCs. The initial locations of the CTCs are given by the function *f* (*x*). The diffusion coefficient in Eq. 4 is denoted by *v*. Equation 4 are the Navier–Stokes equations representing the background plasma, which at this level of model approximation we treat as an incompressible Newtonian fluid. The flow takes place in the upper-half space, above a solid wall which models the vessel wall, hence boundary conditions for the concentration field and blood velocity at the wall are:

(6) $$\frac{\partial \vec{c}}{\partial n}{|}_{\text{wall}}=0$$

(7) $$\vec{u}{|}_{\text{wall}}=0$$

The first is a no penetration condition for the concentration field, while the second is the viscous no slip boundary condition at the wall.

### The green’s function approach

In this paper, we focus on the simple geometry associated with the upper-half plane in 2D and upper-half space in 3D, making it possible to use an analytical Green’s function formulation to form solutions that satisfy exact boundary conditions. In 2D, with no flow (*u* = 0), we use the standard Green’s function associated with the 2D diffusion equation (Kevorkian, 1989):

$$\begin{array}{rc} c^{\left(i\right)}\left(x,y;\;t\right)=\frac{\Gamma }{\sqrt{4\pi vt}}\exp\left(\frac{-{\left(x-x_{i}\right)}^{2}-{\left(y-y_{i}\right)}^{2}}{4vt}\right)\\ c_{\text{image}}^{\left(i\right)}\left(x,y;\;t\right)=\frac{\Gamma }{\sqrt{4\pi vt}}\exp\left(\frac{-{\left(x-x_{i}\right)}^{2}-{\left(y+y_{i}\right)}^{2}}{4vt}\right) & \text{(8)} \end{array}$$

Here, each particle is placed in the upper-half plane (*y *> 0), at positions (*x_i_*, *y_i_*) for *i* = 1, …, *n*, and image particles are placed at (*x_i_*, −*y_i_*). The no penetration condition Eq. 6 for the concentration field at the wall is enforced exactly, with no other explicit boundary conditions needed.

In 3D, with flow *u* = constant, the corresponding Green’s function is given by Kevorkian (1989), Stockie (2011):

$$\begin{array}{ll} c\left(r,x_{i},y_{i},z_{i};\;t\right)=\frac{Q_{\text{T}}}{8{\left(\pi r\right)}^{3∕2}}\exp\left(-\frac{{\left(x_{i}-ut\right)}^{2}+y_{i}^{2}}{4r}\right)\\ \;\times \left[\;\exp\;\left(-\frac{{\left(z_{i}-H\right)}^{2}}{4r}\right)+\exp\;\left(-\frac{{\left(z_{i}+H\right)}^{2}}{4r}\right)\;\right] & \text{(9)} \end{array}$$

Here, $r=\alpha \;x_{i}∕u,$ is a new scaled independent variable. The symbol *Q*_T_ expresses the total amount of TF expressed by the CTC.

Using these solutions as the basic building blocks for flow simulations based on Eqs 1–7, we are able to perform highly resolved concentration-flow simulations described in Section “Results.”

### Concentration field gradient tracking diagnostics

Since the concentration field is fairly complex, we need a diagnostic tool to help with visualization during a simulated run. It is useful to use what we call “passive gradient trackers,” which are diagnostic particles placed in the flow. The gradient trackers do not disturb the flow, but move toward regions of high TF concentration and low TF concentration in time, as the simulation proceeds. A schematic of one of these trackers is shown in the upper left of Figure 3. If the tracker is placed at position (*x*, *y*, *z*) at time “*t*,” the concentration field at that point is given by $\vec{c}\left(x,y,z;\;t\right).$ The tracker then measures the concentration field at six neighboring points in the field: $\vec{c}\left(x\;\pm \varepsilon ,y\pm \varepsilon ,z\pm \varepsilon ,\;t\right),0<\varepsilon <<1,$ and measures the differences in concentration at these six points compared to the concentration at $\vec{c}\left(x,y,z;\;t\right).$ Thus, it measures the quantities $\left(\vec{c}\left(x,y,z;\;t\right)-\vec{c}\left(x\pm \varepsilon ,y,z;\;t\right)\right),$ $\left(\vec{c}\left(x,y,z;\;t\right)-\vec{c}\left(x-\varepsilon ,y,z;\;t\right)\right),$ $\left(\vec{c}\left(x,y,z;\;t\right)-\vec{c}\left(x,y+\varepsilon ,z;\;t\right)\right),$ $\left(\vec{c}\left(x,y,z;\;t\right)-\vec{c}\left(x,y-\varepsilon ,z;\;t\right)\right),$ $\left(\vec{c}\left(x,y,z;\;t\right)-\vec{c}\left(x,y,z+\varepsilon ;\;t\right)\right),$ $\left(\vec{c}\left(x,y,z;\;t\right)-\vec{c}\left(x,y,z-\varepsilon ;\;t\right)\right),$ and if it is seeking high concentration regions, it moves to the point yielding the largest increase in concentration. If it seeks low concentrations, it moves to the point yielding the largest decrease. Thus, it tracks “gradients” in the concentration field at each time step, and as time evolves, the particles will gather in high/low TF concentration regions giving a useful visual diagnostic tool. For our simulations, we use “red” trackers to follow increases in gradient, and “blue” trackers to follow decreases. We note that there is an inherent timescale associated with the tracking, which is essentially governed by the size of ε. In the limit as this parameter goes to zero, the discrete trackers approximate derivatives in concentrations, hence gradients.

## Results

### Two-dimensional concentration fields

**Figure 1:**
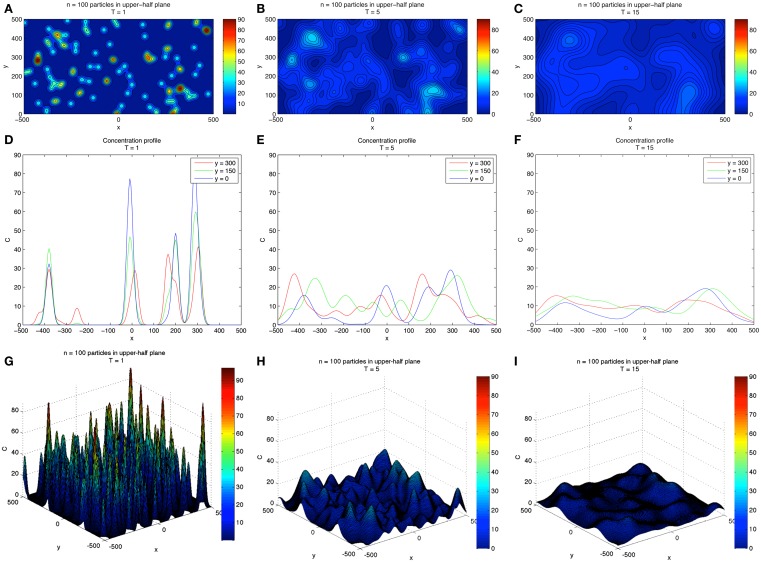
**Two-dimensional concentration fields for 100 diffusing CTCs**. **(A–C)** Show evolving concentration fields at *T* = 1, 5, 15. **(D–F)** Show concentration profiles at different *y*-slices: *y* = 0, 150, 300. Vessel wall is located at *y* = 0. **(G–I)** Show 3D surface plot of the concentration fields and gives a sense of how the field diffuses in time. Boundary conditions are discussed in text.

**Figure 2:**
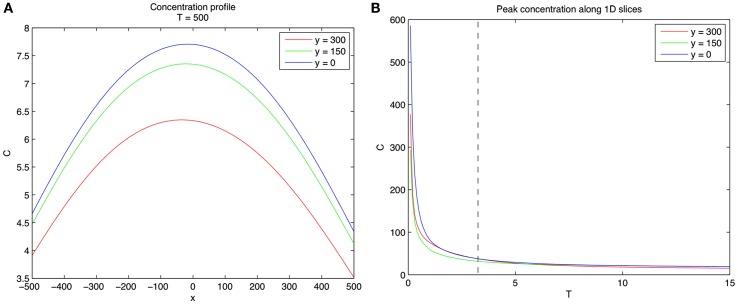
**Concentration profiles for 100 diffusing CTCs**. **(A)** Concentration profiles at *y* = 0, 150, 300 for *T* = 500. Note that concentration is highest near wall. **(B)** Concentration profiles as a function of *T* for *y* = 0, 150, 300. Vertical dashed line separates two distinct regimes. Left of the dashed line (0 < *T *< 3) represents the fast mixing regime of the concentration fields due to the different CTCs, whereas right of the dashed line (*T *> 3) the fields from different CTCs have mixed and are slowly diffusing.

A two-dimensional simulation of developing concentration gradients for 100 diffusing CTCs with no flow (*u* = 0) is shown in Figure 1. Figures 1A–C shows the concentration field at times *T *= 1, 5, 15 with *u* = 0 in Eq. 1. The CTCs are randomly placed in the upper-half plane (*y *> 0), with the vessel wall at *y* = 0. On the vessel wall, we use the no penetration condition Eq. 6 for the concentration field. No other explicit boundary conditions are needed when using the Green’s functions formulas. Figures 1D–F shows the concentration profiles at *y* = 0, 150, 300, while Figures 1G–I shows the 3D surface plots of the concentration fields in the (*x*, *y*) plane. The CTCs are placed in the region *y *> 0, while their images are placed appropriately at *y *< 0 (see Eq. 8) so that boundary conditions are enforced. The (dimensionless) diffusion coefficient for each particle is taken to be α*_i_ *= 1.5. We note that here, and in all of the following simulations, equations, and parameters are to be interpreted non-dimensionally since explicit comparisons with *in vivo* experiments are not described in this paper. The 2D simulations with no flow clearly show the diffusing fields from each particle merging and smoothing over time, with concentration persisting at the vessel wall because of the no penetration boundary condition. This is seen most clearly in Figure 2 which shows the concentration profile for *T *= 500 at *y *= 0, 150, 300. Figure 2A shows the persistence of the highest concentration at the wall (*y *= 0). Figure 2B shows the peak concentration at *y *= 0, 150, 300 as time progresses. The vertical line in this figure separates two distinct temporal regimes: (i) 0 < *T *< 3; (ii) *T *> 3. The first early regime represents a “rapid mixing” regime where the concentration fields quickly merge to form a complex combined overlap domain of fields associated with the different sources merging together. The “long-time” regime (*T *> 3) shows that the peak combined concentration field continues to decay, but rather slowly, with the peak wall concentration (*y* = 0) dominating.

### Three-dimensional concentration fields

**Figure 3:**
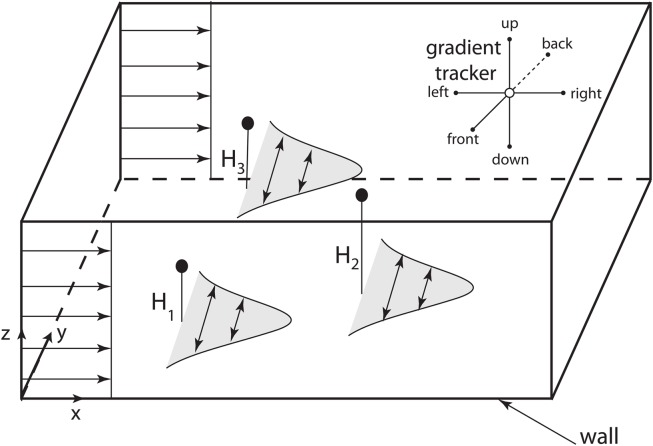
**Computational domain for 3D model of diffusing CTCs**. Schematic shows 3D computational domain with bottom vessel wall at *z* = 0. CTCs are initially placed at random initial positions and travel downstream with the flow, which is constant. The concentration fields diffuse as “puffs,” to model the diffusing TF which initially coats the outer cell surface. Upper right corner of the domain shows the six directions that are “polled” by gradient trackers placed in the flow field.

We next performed a high resolution (exact, since we are using the Green’s function formulation) simulation of CTCs in three-dimensions with a constant flow velocity profile (*u* = constant). Figure 3 shows the general schematic diagram with flow only in the direction of *x*, with diffusing CTCs initially placed in the domain at random heights *z* = *H*_1_, *H*_2_, *H*_3_. The vessel wall in these simulations is located at *z* = 0. Because the concentration fields are spatially complex and time-dependent, we build in particle gradient tracking capability in our code, also shown schematically in Figure 3.

**Figure 4:**
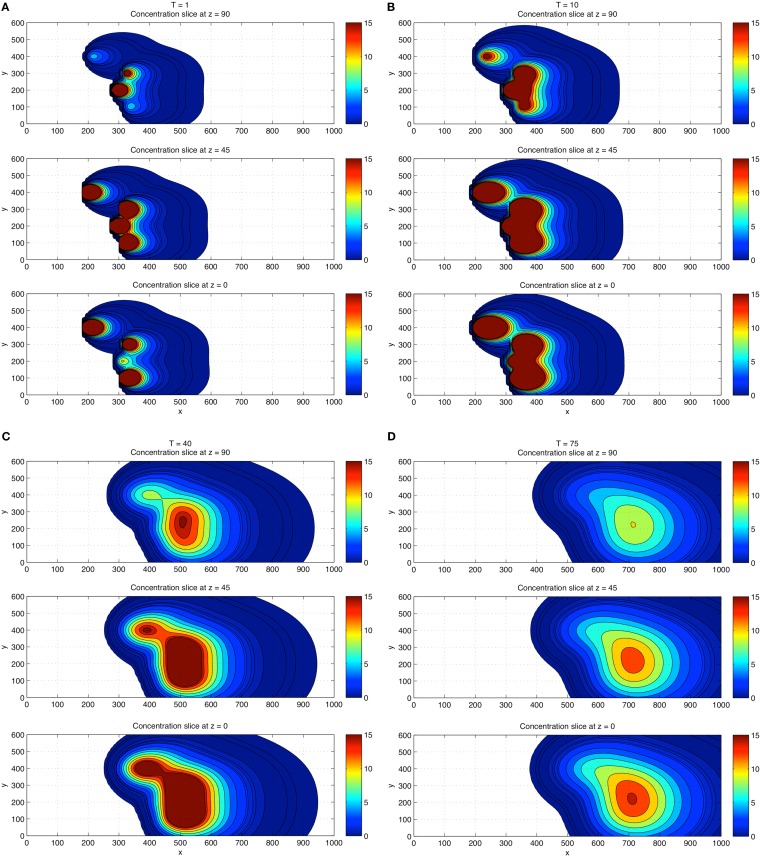
**Three-dimensional concentration field simulations using four CTCs**. View is top down. Top down view of the *x*–*y* plane with four CTCs placed randomly in the flow. Three different *z*-slices are shown: *z* = 0 (wall), *z* = 45, *z* = 90 at each time *T* = 1 **(A)**, *T* = 10 **(B)**, *T* = 40 **(C)**, and *T* = 75 **(D)**. Coloring shows that fields persist and are strongest near the wall region and in overlap domains from different CTCs.

Next, we performed a 3D concentration field simulation (without gradient trackers) using four CTCs, where we show a top down *z*-projection view of the (*x*, *y*) plane for values *z* = 0 (wall), *z* = 45, and *z* = 90, progressively in time *T* = 1, 10, 40, 75 in Figures 4A–D. In order to compare with the 2D simulations, we have chosen the same dimensionless diffusion coefficient values α*_i_ *= 1.5. For these simulations, the initial locations of the particles are (*x*, *y*, *z*) = (300, 300, 45); (180, 400, 30); (300, 100, 30); (275, 200, 60). Careful examination of the coloring of the fields indicates (i) the persistence of the strongest concentration region near the wall, (ii) strong concentrations in overlap domains from different CTCs, and (iii) strong concentrations near each of the CTCs which express TF. These numbers and results are consistent with the experiment described in Tormoen et al. (2011) in which small numbers TF-coated micro-spheres were placed in blood solution and clotting time was carefully measured. Typically, in metastatic patients, measured numbers of CTCs would be in the range of 1–100 CTCs/ml.

**Figure 5:**
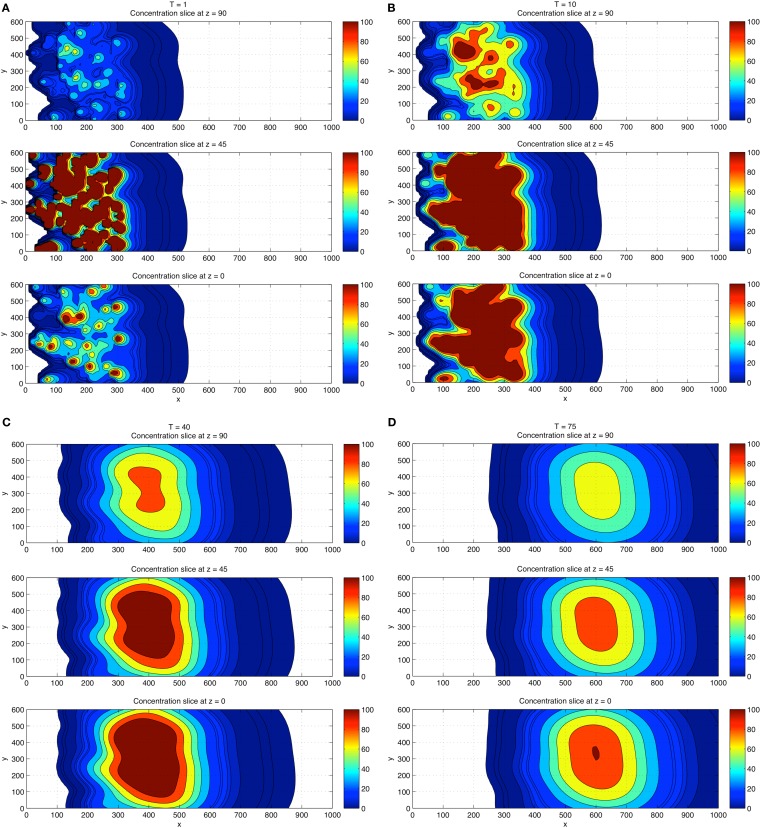
**Three-dimensional concentration field simulations using 100 CTCs**. View is top down. Top down view of the *x*–*y* plane with four CTCs placed randomly in the flow. Three different *z*-slices are shown: *z* = 0 (wall), *z* = 45, *z* = 90 at each time *T* = 1 **(A)**, *T* = 10 **(B)**, and *T* = 40 **(C)**, and *T* = 75 **(D)**. Coloring shows that fields persist and are strongest near the wall region and in overlap domains from different CTCs. Comparisons of the concentration field levels with those in Figure 4 indicate that the concentration field varies (roughly) linearly with the number of CTCs in the domain.

For a direct comparison with the 2D results, we show in Figures 5A–D the 3D concentration field simulations using 100 CTCs placed randomly (initially). The overall concentration field again persists near the wall, but the overall concentration field level is higher, roughly increasing linearly with the number of CTCs in the flow, also in agreement with results from Tormoen et al. (2011). As the flow progresses in time, flow visualization tools become crucial to help understand the field patterns that develop.

### Gradient tracking results

**Figure 6:**
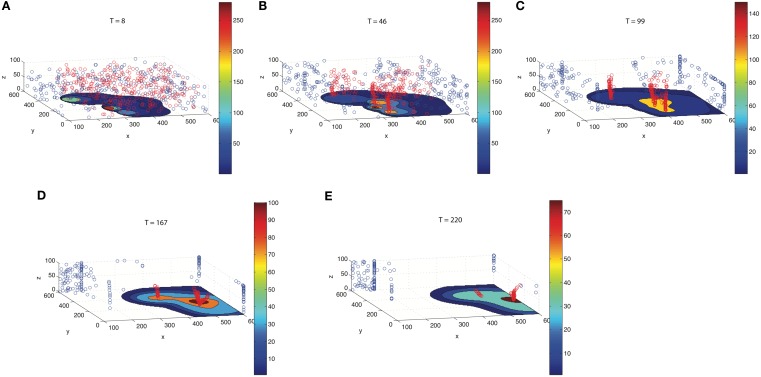
**Gradient trackers which move to regions of high concentration (red) and low concentration (blue)**. As time progresses [**(A)** *T* = 8; **(B)** *T* = 46; **(C)** *T *= 99; **(D)** *T* = 167; **(E)** *T* = 220], red gradient trackers move to regions near the CTCs, then toward regions where the fields from different CTCs overlap, and finally toward the vessel wall where the concentration fields persist. Blue gradient trackers move to regions of low TF concentration.

To track developing TF concentration gradient patterns, we include gradient tracking capability to our simulation. Figure 6 illustrates different time points (*T* = 8–220 s) as the flow progresses. The red particles move toward regions of high CTC concentration, whereas the blue move to regions of low CTC concentration. The patterns that develop with the red and blue particles depend on the comparison of relative timescales as determined by the concentration field diffusion rates, α*_i_*, as well as the timescales on which the gradient trackers move. The first three panels in the figure clearly show the red gradient trackers gathering in highly concentrated regions near each of the CTCs, lining up in elongated columnar strands. The timescale in which these trackers locate the diffusing CTCs is *short* compared with the timescales on which the diffusion fields spread. The last two figures in the panel show the particles then moving toward the vessel wall where concentration fields persist. This movement of the trackers to the vessel walls takes place on a longer timescale, well after the CTCs have been located in the flow.

## Discussion

In this paper, we develop novel computational tools to model and characterize the movement of diffusing thrombin fields emitted from CTCs in flow, with the aim of scaling up the techniques to more complex arterial environments and more complex, time-dependent flow assumptions (Pedley, 1980). A main finding from our model is the build-up and persistence of thrombin concentration near vessel walls and in complex time-dependent overlap regions of the flow. The build-up near walls occurs on a relatively long timescale compared to the timescale in which the concentration fields diffuse (regulated by the diffusion constants α*_i_*). We expect this main finding to persist under more complex and realistic flow geometries, as locally, near a vessel wall, the boundary curvature should not play a big role. Furthermore, very near the vessel wall where viscous flow boundary conditions dominate, blood velocity magnitudes are small, so should have minimal effects on disturbing the concentration fields that build-up there, therefore we do not expect the inviscid fluid boundary conditions used in this study to qualitatively alter this main finding. In complex capillary beds where branching of the flow into several different regions is the reality, we do expect the tracking of the diffusive overlap regions of many CTCs to be more computationally challenging, yet the main finding that concentration levels are relatively high in overlap regions should remain valid. Thrombin has several well characterized effects on endothelial cells and platelets which are also located at blood vessel walls under flow. In addition, the proximity of CTCs to each other determined the persistence of thrombin concentrations in the flow, which may help explain the effect of cell count on coagulation kinetics (Tormoen et al., 2011), suggesting that CTC counts may hold significance to understanding the role for CTCs in activating blood coagulation. The gradient tracking capability described here holds strong potential to aid in the understanding of the fate of CTC-generated thrombin in more complex settings that arise within the circulation.

## Conflict of Interest Statement

The authors declare that the research was conducted in the absence of any commercial or financial relationships that could be construed as a potential conflict of interest.

## References

[B1] Amirkhosravi A. Meyer T. Chang J. Y. Amaya M. Siddiqui F. Desai H. Francis J. L. (2002). Tissue factor pathway inhibitor reduces experimental lung metastasis of B16 melanoma. Thromb. Haemost. 8, 930–936 12083498

[B2] Berny-Lang M. A. Aslan J. E. Tormoen G. W. Patel I. A. Bock P. E. Gruber A. McCarty O. J. (2011). Promotion of experimental thrombus formation by the procoagulant activity of breast cancer cells. Phys. Biol. 8, 015014 10.1088/1478-3975/8/1/015014 21301066 PMC3209705

[B3] Blom J. W. Doggen C. J. Osanto S. Rosendaal F. R. (2005). Malignancies, prothrombotic mutations, and the risk of venous thrombosis. JAMA 293, 715–722 10.1001/jama.293.6.715 15701913

[B4] Bodnar T. Sequeira A. (2008). Numerical simulation of the coagulation dynamics of blood. Comput. Math. Methods Med. 9, 83–104 10.1080/17486700701852784

[B5] Chatterjee M. S. Denney W. S. Jing H. Diamond S. L. (2010). Systems biology of coagulation initiation: kinetics of thrombingeneration in resting and activated human blood. PLoS Comput. Biol. 6, e1000950 10.1371/journal.pcbi.1000950 20941387 PMC2947981

[B6] Fogelson A. L. (2007). “Cell-based models of blood clotting,” in Single Cell-Based Models in Biology and Medicine, eds Anderson A. R. A. Chaplain M. A. J. Rejniak K. A. (Basel: Birkhauser Verlag), 243–269

[B7] Fogelson A. L. Tania N. (2005). Coagulation under flow: the influence of flow-mediated transport on the initiation and inhibition of coagulation. Pathophysiol. Haemost. Thromb. 34, 91–108 10.1159/000089930 16432311

[B8] Gay F. J. Felding-Habermann B. (2011). Contribution of platelets to tumor metastasis. Nat. Rev. Cancer 11, 123–134 10.1038/nrc3004 21258396 PMC6894505

[B9] Gemmell C. H. Turitto V. T. Nemerson Y. (1988). Flow as a regulator of the activation of factor X by tissue factor. Blood 72, 1404–1406 3262388

[B10] Gomez K. McVey J. H. (2006). Tissue factor initiated blood coagulation. Front. Biosci. 11, 1349–1359 10.2741/1888 16368521

[B11] Gregg K. L. (2010). A Mathematical Model of Blood Coagulation and Platelet Deposition Under Flow. Ph.D. thesis, University of Utah, Salt Lake City

[B12] Hall C. L. Taubman M. B. Nemerson Y. Turitto V. T. (1998). Factor Xa generation at the surface of cultured rat vascular smooth muscle cells in an in vitro flow system. J. Biomech. Eng. 120, 484–490 10.1115/1.2798018 10412419

[B13] Kevorkian J. (1989). Partial Differential Equations: Analytical Solution Techniques. Pacific Grove, CA: Wadsworth & Brooks/Cole Mathematics Series

[B14] Khorana A. A. Francis C. W. Culakova E. Kuderer N. M. Lyman G. H. (2007). Thromboembolism is a leading cause of death in cancer patients receiving outpatient chemotherapy. J. Thromb. Haemost. 5, 632–634 10.1111/j.1538-7836.2007.02374.x 17319909

[B15] Leiderman K. Fogelson A. L. (2011). Grow with the flow: a spatial-temporal model of platelet deposition and blood coagulation under flow. Math. Med. Biol. 28, 47–84 10.1093/imammb/dqq005 20439306 PMC3499081

[B16] Marchetti M. Diani E. Ten Cate H. Falanga A. (2012). Characterization of thrombin generation potential of leukemic and solid tumor cells by the calibrated automated thrombography. Haematologica 97, 1173–1180 10.3324/haematol.2011.055343 22419573 PMC3409814

[B17] Marchetti M. Russo L. Balducci D. Falanga A. (2011). All trans-retinoic acid modulates the procoagulant activity of human breast cancer cells. Thromb. Res. 128, 368–374 10.1016/j.thromres.2011.03.006 21458031

[B18] McGee M. P. Li L. C. Xiong H. (1992). Diffusion control in blood coagulation. Activation of factor X by factors IXa/VIIIa assembled on human monocyte membranes. J. Biol. Chem. 267, 24333–24339 1447184

[B19] Mueller B. M. Reisfeld R. A. Edgington T. S. Ruf W. (1992). Expression of tissue factor by melanoma cells promotes efficient hematogenous metastasis. Proc. Natl. Acad. Sci. U.S.A. 89, 11832–11836 10.1073/pnas.89.24.11832 1465406 PMC50651

[B20] Mueller B. M. Ruf W. (1998). Requirement for binding of catalytically active factor VIIa in tissue factor dependent experimental metastasis. J. Clin. Invest. 101, 1372–1378 10.1172/JCI930 9525979 PMC508714

[B21] Nemerson Y. (1968). The phospholipid requirement of tissue factor in blood coagulation. J. Clin. Invest. 47, 72–80 10.1172/JCI105716 16695947 PMC297149

[B22] Okorie U. M. Denney W. S. Chatterjee M. S. Neeves K. B. Diamond S. L. (2008). Determination of surface tissue factor thresholds that trigger coagulation at venous and arterial shear rates: amplification of 100 fM circulating tissue factor requires flow. Blood 111, 3507–3513 10.1182/blood-2007-08-106229 18203955 PMC3321613

[B23] Otero L. L. Alonso D. F. Castro M. Cinat G. Gabri M. R. Gomez D. E. (2011). Tissue factor as a novel marker for detection of circulating cancer cells. Biomarkers 16, 58–64 10.3109/1354750X.2010.533282 21128872

[B24] Pedley T. J. (1980). The Fluid Mechanics of Large Blood Vessels. Cambridge Monographs on Mechanics and Applied Mathematics. Cambridge: Cambridge University Press

[B25] Saito Y. Hashimoto Y. Kuroda J. Yasunaga M. Koga Y. Takahashi A. Matsumura Y. (2011). The inhibition of pancreatic cancer invasion-metastasis cascade in both cellular signal and blood coagulation cascade of tissue factor by its neutralization antibody. Eur. J. Cancer 47, 2230–2239 10.1016/S0959-8049(11)70886-3 21621407

[B26] Stockie J. M. (2011). The mathematics of atmospheric dispersion modeling. SIAM Rev. Soc. Ind. Appl. Math. 53, 349–372

[B27] Tormoen G. W. Rugonyi S. Gruber A. McCarty O. J. (2011). The role of carrier number on the procoagulant activity of tissue factor in blood and plasma. Phys. Biol. 8, 066005 10.1088/1478-3975/8/6/066005 22048420 PMC3529913

[B28] Versteeg H. H. Spek C. A. Peppelenbosch M. P. Richel D. J. (2004). Tissue factor and cancer metastasis: the role of intracellular and extracellular signaling pathways. Mol. Med. 10, 6–11 10.1007/s00894-003-0157-6 15502877 PMC1431349

[B29] Welsh J. Smith J. D. Yates K. R. Greenman J. Maraveyas A. Madden L. A. (2012). Tissue factor expression determines tumor cell coagulation kinetics. Int. J. Lab. Hematol. 34, 396–402 10.1111/j.1751-553X.2012.01409.x 22348286

[B30] Yates K. R. Welsh J. Echrish H. H. Greenman J. Maraveyas A. Madden L. A. (2011). Pancreatic cancer cell and microparticle procoagulant surface characterization: involvement of membrane-expressed tissue factor, phosphatidylserine and phosphatidylethanolamine. Blood Coagul. Fibrinolysis 8, 680–687 10.1097/MBC.0b013e32834ad7bc 21941170

